# Percutaneous nephrolithotomy or flexible ureteral lithotripsy, which one is better for patients with upper ureteral calculi of 1.5–2.0 cm in diameter

**DOI:** 10.1186/s12894-024-01480-1

**Published:** 2024-04-25

**Authors:** Wenpu Chen, Hengda Hu, Guofeng Yu

**Affiliations:** Department of Urology, Jinshan District Central Hospital affiliated to Shanghai University of Medicine & Health Sciences; Jinshan Branch of Shanghai Sixth People’s Hospital, 147 Jiankang Road, Jinshan District, Shanghai, China

**Keywords:** Percutaneous nephrolithotomy, Flexible ureteral lithotripsy, Upper ureteral stone

## Abstract

**Purpose:**

First research to evaluate the clinical efficacy and safety of flexible ureteral lithotripsy (FURSL) and percutaneous nephrolithotomy (PCNL) in the treatment of the upper ureteral stone is between 1.5 cm and 2.0 cm in diameter since there is no consensus with such ureteral stone yet.

**Methods:**

From December 2018 to October 2022, 104 patients with calculi in the upper ureter received percutaneous nephrolithotomy (PCNL)or flexible ureteroscopic lithotripsy (FURSL) in our institution. The clinical data of the patients in the two groups were retrospectively searched. Stone removal rate, operation time, blood loss, postoperative pain score, postoperative inflammatory factor, postoperative complication rates and ureteral obstruction three months after the operation were compared between the two groups.

**Results:**

A total of 104 patients were included in the study. The stone clearance rate and the secondary surgery rate were 88.89% and 7.41% in the FURSL group, the figures were 97.96% and 2.0% in the PCNL group (*p* = 0.067, 0.497). Regarding ureteral obstruction three months after the operation, there were 2 patients in FURSL group and 0 patients in PCNL group(*p* = 0.497).Compared to patients in FURSL group, patients in the PCNL group had shorter operation time(PCNL 71.81 ± 18.94 min vs. FURSL 86.80 ± 22.49 min, *p* = 0.0004), fewer complications(PCNL 20.37% vs. FURSL 6.12%), and lower postoperative inflammatory factor(*p* = 0.0004), yet they got more hemoglobin drop (PCNL 13.14 ± 9.81 g/L vs. FURSL 4.77 ± 3.55 g/L, *p* < 0.0001), higher postoperative pain scores(*p* = 0.0017) in the first three postoperative days and longer hospital stay (PCNL 4.96 ± 1.21 days vs. FURSL 3.60 ± 0.83 days).

**Conclusion:**

Both FURSL and PCNL were effective methods for treating upper ureteral stones of 1.5–2.0 cm in diameter given the extremely high stone clearance rate and a very low secondary surgery rate, as long as rare ureteral obstruction in medium-long term observation. Additionally, FURSL can effectively reduce surgical bleeding, postoperative pain, and hospital stay, while PCNL can decrease operation time, the risk of infection, and complications. Therefore, doctors could select suitable surgical treatment for those patients depending on their different clinical situations based on these findings.

## Introduction

Urological stones are one of the most common urological disorders in clinical practice for urologists. It reported 1–20% people suffer from urological stones during their lifetime [1, 2]and incidence rate is increasingly rising around the world [3, 4]. The ureteral stones are more severe and often occurred in the upper ureter, as they lead to hydronephrosis and renal impairment [5–7].

To be precise, these upper ureteral stones, usually combining with some degree of hydronephrosis on the affected side are located in the ureter and remain in the same position for days or even weeks and could lead to unilateral renal impairment and severe loss of unilateral renal function. Also, multiple polyp formation below and dilated and tortuous ureters above are associated, which make the treatment harder [8, 9].

In clinical practice, patients could not be cured when only receiving non-operation treatment. Some other surgeries have also been unpopular because of a variety of issues [10, 11]. For example, ureteral rigidoscopy is a complicated procedure for the treatment of stones in the upper ureter and has a low success rate as the stones tend to travel up to the renal pelvis during the surgery. Another option, laparoscopic surgery is indicated for complex and large stones and has been gradually replaced in recent years due to the high degree of trauma and slow postoperative recovery [12–14].Also, extracorporeal shock wave lithotripsy is suitable for patients with upper ureteral stones, the postoperative residual fragments limit its acceptance among patients [15–17].

With the development of minimally invasive urological techniques, the most common and widely-accepted options for upper ureteral calculi are percutaneous nephrolithotripsy (PCNL) and retrograde intrarenal surgery, which is flexible ureteral lithotripsy (FUSRL) in this case. PCNL is recommended for larger upper urinary tract stones, while FURSL is suitable for smaller ones. However, in newest consensus on PCNL [18], which were made by hundreds of experts from Europe and Asia, PCNL is recommended when stones are greater than 1.5 cm in diameter in the upper ureter. While retrograde intrarenal surgery, mainly shown as FURSL is recommended for stones that are smaller than 2.0 cm in diameter in another new consensus by similar experts [19]. Consequently, there is no consensus when the upper ureteral stone is between 1.5 cm and 2.0 cm in diameter.

Thus, we conducted this retrospective study to evaluate the clinical outcomes of PCNL versus FURSL in the treatment of upper ureteral stones by searching and analyzing the clinical data from patients through imaging system in our hospital. To our knowledge, this is the first study regarding upper ureteral stone with a diameter of 1.5 cm to 2.0 cm.

## Patients and method

### Research ethics board approval

This study was approved by the ethics committee of Shanghai Jinshan District Central Hospital (Number: JSZXYY2024007001). All methods were performed in accordance with the Declaration of Helsinki. Informed consent was obtained from all subjects and/or their legal guardians.

### Clinical information

All the patients were retrospectively selected from our institution: Shanghai Jinshan District Central Hospital. The diameter of ureteral stones was measured from coronal, cross-sectional and sagittal CT images, the longest diameter was recorded as the selection basis. patients with upper ureteral stones with a diameter of 1.5 cm to 2.0 cm who were treated by PCNL or FURSL from December 2018 to October 2022, were selected through the imaging system in our institution.

#### Inclusion criteria

(1) the stone was in the upper ureter and located there more than 2 months; (2) clinical data were kept intact; (3) the operators were part of the same surgical team.

#### Exclusion criteria

(1) patients with severe infections such as septic kidney; (2) patients with urological tumors; (3) patients with psychiatric disorders and impaired consciousness; (4) patients with cardiac, pulmonary, hepatic and renal insufficiency; (5) patients with coagulation disorders; (6) patient with cardiovascular and cerebrovascular diseases; (7) with spinal deformities.

### Treatment procedure for PCNL and FURSL

#### Surgical procedure for PCNL group

After receiving general anesthesia with supine lithotomy, patients were inserted with ureteral catheter through the F12 nephroscope(Richard_Wolf)in their upper ureter. Then patients were later changed to the prone position. Subsequently, the patient’s renal area and ureter were scanned to the locate the stone distribution in the ultrasound image using a Danish BK ultrasound detector. Next, a 16G puncture needle (Cook Medical) was passed through the renal papillae of posterior upper or middle calyx of the kidney. Through the urine drainage path, the guidewire (Cook Medical) was placed into the collecting system and the percutaneous tract was formed with a 0.8 cm incision and a fascial dilator dilated from F8 to F18. After this, a holmium laser lithotripter (Lumenis) was attached close to the stone which was subsequently crushed and flushed out of the body with saline. Usually, A 200-micron fiber is typically employed with an initial energy setting of 1.5 joules and a power of 15 W, adjustable up to a maximum of 2.5 J and 25 W. (A 365-micron fiber can also be used to increase the speed.) In cases where the puncture angle or the low position of the ureteral stone makes it challenging to use a nephroscope for lithotripsy, a ureteral flexible scope may be used as a substitute for nephroscopy. Finally, a ureteral stent is smoothly inserted for 2 weeks, and a nephrostomy tube is left for 1 week.

#### Surgical procedure for FURSL group

FURSL group: patients received general anesthesia and were placed in the lithotomy position. An F7.8 ureteroscope (Karl Storz) was inserted through the urethra under direct vision, if there was a double J tube, it should be removed at first. It was followed by the progress that the rigid ureteroscope was removed, the flexible ureteroscope sheath (F12/14, Cook Medical) was inserted through the smooth guide wire and the flexible ureteroscope (F7.8, Karl Storz) was inserted through the sheath. Next, the stone was transferred to the middle or upper calyx of the kidney with a reticular basket and subsequently crushed with holmium laser (Lumenis, 200-micron, from 15 W,1.5 J to 20 W,2.0 J). If the stone is tightly lodged in the ureter, it is advisable to use laser lithotripsy to appropriately fragment the stone(15 W or lower), followed by the use of a guidewire to advance into the renal pelvis. Finally, it was removed out of body with a reticular basket and a double J tube was left in place after the procedure.

After the surgery (PCNL or FURSL), the patient receives antibiotics, and if there was an infection, the patient continued to receive sensitive antibiotics for about 3 days according to the urine culture results, and the blood tests are repeated within 3 days after the surgery. Two weeks after surgery, the patient receiving CT scan detecting residual stones and a removal of the double J-tube, and if there were residual stones, they would get a second operation one month after the surgery.

### Observation index

The following data were collected: Operation time; intraoperative bleeding, measured with hemoglobin drop; stone clearance rate(< 3 mm, measured on post-operative images), measured with CT images before and three month after stent removing surgery; Complication (including high fever, greater than 39℃, ureteral stricture, etc.); secondary surgery rate; Postoperative VAS score (day1 to day3); postoperative inflammatory factor indicators (C-reactive protein, day1 to day3).

### Statistical methods

Data analysis was performed using GraphPad Prism 9 or SPSS 26.0 for Windows. Categorical data were examined using the Chi-square test and the continuous variable was assessed using the independent sample t-test. *p* < 0.05 was considered to indicate a statistically significant difference, and the measurement data of normal distribution were represented by (x ± s) and data were compared using a t-test. Also, data were expressed as percentages (%) or the number of cases.

## Results

### Clinical data comparison between PCNL and FURSL group

A total of 104 patients with upper ureteral stones with a diameter of 1.5 cm to 2.0 cm were included and analyzed in this study. In FURSL group, there were 34 males and 21 females, age ranging from 28 to 77(mean age :55.02 ± 13.43), and in the PCNL group, there were 30 males and 19 females, aged 31 to 74 years (mean age :53.12 ± 9.64), The differences in gender, age, stone diameter, stone density, and hydronephrosis between the two groups were not statistically significant. The degree of hydronephrosis was analyzed as mild/moderate or severe, and there was no statistically significant difference observed between the FRUSL and PCNL groups(*p* = 0.077). (Table 1)

There were 7 patients in FURSL group and 4 patients in PCNL group receiving stent insertion beforehand and no statically significance could be found regarding operation time and complication when compared to those non pre-stent patients in both groups.

### Comparison of perioperative and postoperative-related indexes

PCNL group showed a better performance in operation time and complications (71.81 ± 18.94 min vs. 86.80 ± 22.49 min, *p* = 0.0004; 11 cases (20.37%) vs. 3 cases (6.12%)). FURSL group had a lower Hemoglobin drop (4.77 ± 3.55 g/L vs. 13.14 ± 9.81 g/L, *p* < 0.0001). The postoperative complications were mainly high fever and postoperative bleeding. Eight patients had postoperative high fever in FURSL group while only 2 patients had this in PCNL group. However, one patient in PCNL group had uncontrollable postoperative bleeding and ultimately underwent highly selective embolization. The detailed complication can be seen in Table 2.

The rate of stone clearance and secondary surgery rate between the FURSL group and PCNL group were 88.89% versus 97.96% (*p* = 0.067) and 7.41% versus 2.00%(*p* = 0.420). Four patients in FURSL group received a second surgery to remove the residual stone and the other 2 expulsed stone without a surgery. While in PCNL group, only one patient received super selective renal artery embolization, other patients with residual stone did not need a surgery. Regarding ureteral obstruction (ureteral stricture) three months after the operation, there were 2 patients in FURSL group and 0 patients in PCNL group(*p* = 0.497), these 2 patients received ureteral stenting with 2 stents or balloon dilation subsequently. There is no statistically significant difference in rate of stone clearance, secondary surgery rate and ureteral obstruction three months after the operation. Hospital stay witnessed a statistically significant difference between FURSL group and PCNL group, with 3.60 ± 0.83 and 4.96 ± 1.21 days(*p* = 0.008),respectively.

### Comparison of postoperative pain scores between the two groups in the first three postoperative days

The postoperative pain scores was postoperatively lower in the FURSL group compared to PCNL group (*p* = 0.0017).The scores in FURSL group for the first three postoperative days were 2.46 ± 0.61,1.79 ± 0.54, and 1.30 ± 0.42 respectively and the figures in PCNL group were 3.15 ± 0.68,2.54 ± 0.61, and 1.95 ± 0.58. Comparison of postoperative inflammatory factor indicators between the two groups in the first three postoperative days.

The C-reactive protein gradually decreased significantly in both groups from the 1st to 3rd day postoperative period, while the C-reactive protein higher in FURSL than in PCNL (*p* = 0.0004). (Table 3)

**Table 1:** Clinical data comparison between FURSL and PCNL group

	FURSL (*n* = 55)	PCNL (*n* = 49)	P value
Age(Y)	51.02 ± 10.86	48.26 ± 11.22	0.211
Gender(Male/Female)	34/21	30/19	0.839
CT value	900.64 ± 180.16	864.52 ± 200.83	0.336
Diameter(cm)	1.65 ± 0.31	1.69 ± 0.22	0.376
hydronephrosis	55	49	1
-Mild /moderate hydronephrosis	32	20	0.077
-Severe hydronephrosis	23	29	

**Table 2:** Comparison of Perioperative and Postoperative -Related Indexes

	FURSL	PCNL	P value
Operation time, (min)	86.80 ± 22.49	71.81 ± 18.94	0.0004
Hemoglobin drop (g/L)	4.77 ± 3.55	13.14 ± 9.81	< 0.0001
stone clearance rate	48/55(88.89%)	48/49(97.96%)	0.067
Postoperative complication	11(20.37%)	3(6.12%)	0.035
High fever(>39℃)	8	2	/
Highly selective embolization	0	1	/
Stent dispalcement	2	0	/
Septic shock	0	0	/
transfusion	0	0	/
Perforation	1	0	/
Secondary surgery rate	4/54(7.41%)	1/49(2.00%)	0.420
Ureteral Obstruction 3 months after the operation	2/55(3.64%)	0/49((0%)	0.497
Hospital stay (d)	3.60 ± 0.83	4.96 ± 1.21	0.008

**Table 3:** Comparison of postoperative inflammatory factor between the two groups

	C-reactive protein(mg/L)		
**Postoperative Day**	1	2	3
**FURSL**	8.97 ± 1.35	7.93 ± 1.20	6.80 ± 1.14
**PCNL**	7.32 ± 1.10	6.41 ± 1.03	5.27 ± 1.09

## Discussion

In this retrospective study, 104 patients showed that PCNL and FURSL were both effective methods treating upper ureteral stones of 1.5–2.0 cm in diameter. Patients could get rid of upper ureteral stones after receiving these 2 surgeries, the stone clearance rates were extremely high, 88.89% and 97.96%, respectively. only 7.41%(FURSL) and 2%(PCNL) patients need a second surgery. Furthermore, patients treated with PCNL had higher hemoglobin drop compared to those receiving FURSL, however, they had a shorter operation time, fewer complications and shorter hospital stay. Moreover, in general, patients who undergo FURSL treatment typically only need to stay in the hospital for one day after the operation. However, we were concerned about potential infections in patients with ureteral calculi measuring 1.5–2.0 cm in diameter, so they were kept hospitalized for a longer duration. In clinical practice, these patients will have a significantly shorter hospital stay compared to those undergoing PCNL. 8 patients had postoperative high fever in FURSL group and 2 had high fever in PCNL group. After 1 to 3 days of antibiotic treatment, all these patients had their temperature under control. Also, the postoperative inflammatory factors also showed that PCNL could avoid a possible infection compared to FURSL. Regarding the medium-long term observation, there are more patients in FURSL group with ureteral obstruction three months after the operation, but there is no statistically significant difference.

This study first focused on the upper ureteral stones of 1.5–2.0 cm in diameter although there were plenty of research regarding the treatment of ureteral stones with PCNL or FURSL, as both these 2 surgeries were the most wide-accepted surgeries currently. According to the previous studies, the risk of complications, bleeding, associated with percutaneous nephrolithoscopy, related to its access diameter, upper caliceal puncture, multiple punctures and increased intra-operative time [13–15]. A perfect puncture to avoid bleeding is achieved by creating a concise and direct pathway that starts from the skin, passes through the subcutaneous tissue, and reaches the cup of calyx (renal papillae). This technique effectively safeguards against any potential harm to the anterior or posterior segmental branches [20]. 

In this study, we used 16–18 F sheath guided with ultrasound, and it was reported that postoperative complications are greatly declined with same level of stone-free rate compared to the standard percutaneous nephrolithoscopy [21, 22]. However, the postoperative bleeding issue could not be avoided in practice and highly selective embolization was effective remedy for this problem. In our study, only one patient had postoperative bleeding and received highly selective embolization finally. On the other hand, PCNL is the treatment of choice for large renal stone, especially for staghorn stones. According to the up-to-date consensus on PCNL [11], it is recommended for the stones are greater than 1.5 cm in diameter in the upper ureter.

Meanwhile, ureteral lithotripsy is a procedure that involves the placement of a flexible ureteroscope through the body’s natural lumen, combined with a laser to treat renal or ureteral stones safely and effectively [23, 24]. Ureteral lithotripsy can be adjusted to reach various locations of the renal calyces and effectively remove stones in different locations.Yet it can promote intrarenal pressure results from outflow obstructed by small fragments, as a result, it could increase the risk of infections or even sepsis [25]. Consequently, for these upper ureteral stones, they are pushed into the calyces during surgery at first and then lithotripsy is performed by laser to avoid excessive pressure and damage to the ureter which can cause ureteral stenosis [26]. If the stone is tightly embedded in the ureter, it can be crushed into several pieces with a laser before being pushed into the calyxes.

In our study, the upper ureteral calculi could be displaced to the upper or middle calyces with a mesh basket and then lithotripsy was performed with good results. Another newest consensus on retrograde intrarenal surgery gave a recommendation that a stone less than 20 mm in diameter is the best indication for retrograde intrarenal surgery. Thus, for those 1.5 to 2.0 cm stones in diameter, experts did not give a decisive conclusion according to the newest recommendation. In addition to the expert consensus, we found a few studies comparing PCNL and RIRS for renal or ureteral stones less than 2.0 cm in diameter [27–29]. However, there are no studies that specifically focus on upper ureteral stones of 1.5–2.0 cm in diameter, which means that there is no evidence to prove which procedure is better for stones of this size. Our research has shown that both FURSL and PCNL are effective approaches to treating such stones, each with its own advantages. Although neither FURSL nor PCNL demonstrated decisive superiority, doctors can select the most suitable approach based on our findings, taking multiple factors into consideration.

In this retrospective study, the choice of surgical approach takes into consideration the following factors: the degree of renal hydronephrosis, the distribution of renal peri-vascular structures, especially arteries, the positioning of the intestine, and patient preferences. Since the choice of surgical approach was based on multiple factors, there was no preference for PCNL or FURSL, which somewhat reduced the occurrence of bias. However, the limitation also exists, potential selection bias in this study could not be avoided totally. In addition, the sample size of this study was insufficient and some clinical data were not recorded to draw conclusive conclusions. Therefore, further multicenter randomized controlled trials and randomized controlled trials with larger sample sizes are needed to complement our study.

## Conclusion

Both FURSL and PCNL were effective methods for treating upper ureteral stones of 1.5–2.0 cm in diameter given the extremely high stone clearance rate and a very low secondary surgery rate, as long as rare ureteral obstruction in medium-long term observation. Additionally, FURSL can effectively reduce surgical bleeding, postoperative pain, and hospital stay, while PCNL can decrease operation time, the risk of infection, and complications. Therefore, doctors could select suitable surgical treatment for those patients depending on their different clinical situations based on these findings.

## Data Availability

Data availabilityThe datasets used and/or analyzed during the current study are available from the corresponding author on reasonable request.

## Abbreviations

PCNL: Percutaneous nephrolithotripsy
FUSRL: Flexible ureteral lithotripsy
VAS: Visual Analog Scale
